# How Cheap Is Soaring Flight in Raptors? A Preliminary Investigation in Freely-Flying Vultures

**DOI:** 10.1371/journal.pone.0084887

**Published:** 2014-01-15

**Authors:** Olivier Duriez, Akiko Kato, Clara Tromp, Giacomo Dell'Omo, Alexei L. Vyssotski, François Sarrazin, Yan Ropert-Coudert

**Affiliations:** 1 Centre d'Ecologie Fonctionnelle et Evolutive (UMR5175), Université Montpellier 2, Biogeography and Ecology of Vertebrates, Montpellier, France; 2 Université Pierre et Marie Curie, UMR7204 CERSP, Muséum National d'Histoire Naturelle, Paris, France; 3 Université de Strasbourg, IPHC, Strasbourg, France; 4 CNRS, UMR7178, Strasbourg, France; 5 Ornis italica, Rome, Italy; 6 Institute of Neuroinformatics, University of Zurich/ETH Zurich, Zurich, Switzerland; University of Regina, Canada

## Abstract

Measuring the costs of soaring, gliding and flapping flight in raptors is challenging, but essential for understanding their ecology. Among raptors, vultures are scavengers that have evolved highly efficient soaring-gliding flight techniques to minimize energy costs to find unpredictable food resources. Using electrocardiogram, GPS and accelerometer bio-loggers, we report the heart rate (HR) of captive griffon vultures (*Gyps fulvus* and *G. himalayensis*) trained for freely-flying. HR increased three-fold at take-off (characterized by prolonged flapping flight) and landing (>300 beats-per-minute, (bpm)) compared to baseline levels (80–100 bpm). However, within 10 minutes after the initial flapping phase, HR in soaring/gliding flight dropped to values similar to baseline levels, i.e. slightly lower than theoretically expected. However, the extremely rapid decrease in HR was unexpected, when compared with other marine gliders, such as albatrosses. Weather conditions influenced flight performance and HR was noticeably higher during cloudy compared to sunny conditions when prolonged soaring flight is made easier by thermal ascending air currents. Soaring as a cheap locomotory mode is a crucial adaptation for vultures who spend so long on the wing for wide-ranging movements to find food.

## Introduction

Flying allows birds to travel fast and far, but generally yields high energetic costs per unit time. Wing flapping typically leads to a seven fold increase in metabolic rate above basal (BMR) [1] and the muscular power required for flapping flight increases steeply with body mass [2]. Large birds have thus evolved alternative flight styles, such as soaring where the main propulsive force is the strength of air currents rather than internal muscular energy [3]. Large seabirds, such as albatrosses, perform low cost ‘dynamic soaring flight’ by making using of the energy in vertical wind shears, i. e. the differences in horizontal wind speed according to the altitude above the waves [4]–[6]. Because this technique cannot generally be used above land, large terrestrial birds like raptors or storks gain altitude by circling inside ascending thermal air currents or using orographic uplift (i.e. wind current diverted upwards along a mountain slope), before gliding [7]–[11].

The energetic cost of soaring and gliding is expected to be low (theoretically about 1.5× BMR [1]). However, measures of energy use are difficult in free-ranging individuals. Although metabolism in flight can be precisely monitored by measuring oxygen consumption through oxygen masks while birds fly in a wind tunnel, this method obviously cannot be used on free soaring birds that need space to fly. Another extensively-used approach involves doubly-labelled water [12], [13], but this method only gives average values over several hours to several days, making the comparison of energy expended during various phases of the flight impossible, especially gliding vs. flapping flight [14], [15]. A third way to estimate flight costs of wild birds is to measure heart rate (HR) of individuals as a proxy for energy expenditure. Many studies have reported a correlation between HR and oxygen consumption [16]. The major advantage of this method is that it becomes possible to associate an estimated cost with a specific event, such as soaring-gliding vs. flapping flight, especially when HR is derived from fine-scale electro-cardiogram (ECG) data [17], [18]. HR has been used to evaluate flight costs in several species of seabirds that use gliding or flapping flights [4], [17]–[20], as well as in waterbirds like Anatidae that use flapping flight [14], [21] and white pelicans, *Pelecanus onocrotalus*, that were measured gliding in V-shape formation over water - but did not soar - [22]. In land birds, differences in HR in soaring-gliding flight have been examined in only two species: bee-eaters, *Merops apiaster*, that alternate short bouts of soaring-gliding and flapping flight [23], and one raptor, the Turkey vulture, *Cathartes aura* [24]. Although Turkey Vultures are specialized for soaring-gliding flight, Mandel et al. [24] only reported averaged hourly values of HR (with no reference made to resting values) and did not explicitly distinguish between soaring-gliding and flapping bouts. One of the major limitations that has so far prevented detailed studies of HR in large soaring birds (particularly raptors and unlike seabirds such as albatrosses) is their shy behaviour, the difficulty of catching the birds to deploy the apparatus, and the even more difficult task of catching them again to recover the device and download the data.

Vultures have evolved the most extreme use of soaring-gliding flight [25], [26]. As an illustration, the record of altitude attained by a bird belongs to a Rüppell's vulture, *Gyps ruppellii*, that collided with an airplane at 11,000 m [26]. It is thus understandable that this species has become the focus of studies on soaring-gliding flight [7], [27]. From an ecological perspective, being obligatory scavengers, vultures evolved an extremely opportunistic lifestyle to cope with food resource unpredictable in time and space [28]. Vultures face a trade-off as they need to travel great distances in search of food while minimizing energy expenditure and storing large body reserves [29], [30]. This leads to the prediction that they will exhibit adaptations to limit energy expenditures while in flight.

Here we report the first measurements of HR (derived from electrocardiogram, i.e. offering unparalleled precision in HR recording) recorded together with high-resolution flight behaviour (using GPS and accelerometer) in two species of free flying vultures specialized for prolonged gliding flight: Eurasian griffon vulture, *Gyps fulvus*, and Himalayan griffon vulture, *G. himalayensis*. The fine-scale measurement of flight activity allowed us to compare HR between different phases of flight (soaring, gliding, flapping, take-off and landing) and to relate it to resting when birds are at the nest or walking. We examined the pattern of HR decrease following the intense effort involved in flapping to quantify the importance of flapping for energy budgets. HR in flying albatrosses, marine scavengers facing similar ecological constraints, have been measured at 2× BMR but took up to 2 hours to recover from the intense muscular effort associated with take-off [4], [18]. We expected similar results for soaring vultures. We related HR in flight with weather conditions, particularly solar radiation since this generates ascending currents and can profoundly influence soaring activity [9]–[11]. We hypothesized that vultures would use more flapping flight during cloudy than sunny periods, resulting in higher HR values.

## Materials and Methods

### a) Study site, birds, and experimental setting

The study site (Rocher des Aigles, Rocamadour, France), overhangs a 120 m-deep canyon, providing natural soaring conditions for raptors. Vultures were trained with falconry techniques to execute free flight shows for the public. During recording sessions, birds were released together from their perch. Just before the flight, the trainer approached the birds and remained close to them for a few minutes before they were released. Once free, birds generally took-off immediately. They could freely choose their flight path, presumably to find an ascending current to climb. They were recalled by the trainer after 10–20 min. However, since the timing of the signal of the trainer was not strictly fixed from one show to another and since birds sometimes responded several minutes after this signal, we do not think that the timing modified the flight behaviour of the birds. Birds landed in an open arena and then had to walk 200–300 m back to their perch. Preliminary experiments indicated that the birds flew similar to wild vultures (C. Tromp, unpublished results), reaching heights >1500 m above ground within 15 minutes. We collected data for one Himalayan griffon (age 15) and one Eurasian griffon (age 30) that performed 11 and 12 flights respectively over four days (mean flight duration 11.0±5.2 min, range 3–19 min). These 2 individuals were chosen for their tame behaviour, tolerance of handling and tolerance for a harness and HR data logger, a prerequisite to obtain normal soaring flight behaviour to reach high altitudes. In contrast birds that were stressed with the harness were more prone to make short flights in the vicinity of the platform where the trainer waited. Although belonging to two closely-related species [31], the two individuals were similar in morphometry (Table 1) and are thus expected to face similar constraints for soaring-gliding flight. Weather conditions were recorded at the site on the day of each experiment and for each flight. Two conditions prevailed during experiments: sunny days with no cloud cover and days with 100% cloud cover.

**Table 1:** Morphometry and flight characteristics of the two vultures in different weather conditions. Values are mean ± SD with sample size in brackets.

Species		Himalayan griffon		Eurasian griffon	
Body mass (kg)		7.5		7.7	
Wingspan (m)		2.80		2.56	
Wing area (m^2^)		1.22		0.95	
Wing loading (kg.m^−2^)		6.13		8.12	
Weather		Sunny	Cloudy	Sunny	Cloudy
Flight duration (min)		15.9±4.9 (6)	10.0±1.9 (4)	11.1±4.2 (7)	5.7±3.4 (5)
Maximum flight height (m)		615.7±207.3 (6)	363.7±32.2 (4)	462.8±120.5 (7)	336.5±21.2 (5)
% flight time engaged in:	Flap	4.0±4.2 (6)	11.6±3.8 (4)	2.8±1.5 (7)	5.4±1.5 (5)
	Glide	50.8±13.2 (6)	51.8±12.3 (4)	52.5±7.5 (7)	52.0±5.8 (5)
	Soar	45.2±13.5 (6)	36.6±11.5 (4)	44.7±7.2 (7)	42.6±5.6 (5)
Flight bout duration (sec)	Flap	1.1±0.8 (165)	1.1±0.7 (248)	1.4±0.8 (88)	1.4±0.6 (64)
	Glide	15.9±40.0 (185)	5.3±7.9 (228)	18.3±24.3 (133)	11.0±15.3 (80)
	Soar	34.5±43.3 (76)	18.1±25.1 (51)	31.0±25.8 (68)	36.9±35.6 (20)
Mean HR (bpm)	Flap	267.0±62.5 (165)	280.5±61.5 (248)	265.5±65.3 (88)	247.4±66.4 (64)
	Glide	235.8±84.0 (185)	279.3±65.3 (228)	119.6±95.3 (133)	211.0±81.8 (80)
	Soar	168.4±65.9 (76)	224.7±62.1 (51)	110.7±28.6 (68)	109.9±11.0 (20)
Mean ODBA (mG)	Flap	559.4±165.7 (165)	566.5±129.7 (248)	582.8±200.8 (88)	573.5±184.2 (64)
	Glide	189.7±103.8 (185)	198.6±86.9 (228)	127.6±96.2 (133)	138.1±79.2 (80)
	Soar	109.0±64.5 (76)	161.1±114.0 (51)	87.0±72.7 (68)	67.1±13.8 (20)

### b) Data loggers

Vultures were equipped with bio-loggers including a GPS, a 3-axial accelerometer, and an electrocardiogram recorder (ECG). The 3D positions of birds were recorded 4 times per second using the high-precision GPS module (Gipsy 1, Technosmart, Italy). We performed immobility tests under open sky to assess the precision of the GPS loggers by calculating standard deviation around mean values of longitude, latitude and altitude (respectively 0.8, and 1.7 and 1.2 m). Three-axial accelerations between −2.3 and +2.3 G or between −9.2 and +9.2 G were acquired at 100 Hz with a LIS302DL accelerometer (STMicroelectronics) and stored in Neurologger 2 [32]. The 52 g ECG logger (UWE-ECG, Little Leonardo, Japan) recorded the ECG at 1000 Hz through 3 cables that were clipped in the skin of the vultures' back with golden-plated safety pins [17]. Two electrodes were clipped on each side of the thorax and one on the middle of the back (ground).

The GPS, accelerometer and ECG were taped together and attached to a harness, which consisted of an aluminium plate (100×60 mm) and two Teflon ribbon strips (with silicon tubing for elasticity). The plate was positioned approximately at the centre of gravity on the back of the bird and held by leg-loops [33], [34]. The total mass of the harness and loggers was 215 g which represented <1.5% of total body mass.

### c) Analyses

ECG and acceleration data were processed using a program developed on Igor Pro for this purpose (Wavemetrics, USA). The vectorial Overall Dynamic Body Acceleration (generally called ODBA or VeDBA) was calculated from the acceleration values, allowing identification of periods of inactivity (resting or soaring-gliding) and activity (walking or flapping) [35]–[37]. At first the dynamic acceleration was calculated for each axis by subtracting the smoothed acceleration from total acceleration. Then the ODBA was calculated as a vectorial sum i.e. the square root of the sum of the squares of the dynamic accelerations. To calibrate ODBA with flight behaviour, vultures were observed and filmed after take-off for as long as possible (birds could not be observed 100% of time during flight since they often went out of sight in the canyon), and again while landing. Preliminary analyses on a larger sample of vultures tracked without an ECG logger (C. Tromp, unpublished results) found a strong correlation between 3D acceleration, ODBA and flight behaviour, as has been observed for other flying birds [38]–[41]. The distinction between flapping flight vs. soaring/gliding was evident from vertical and forward acceleration component because the acceleration fluctuated with each wing beat [38] (see Figure S2). We are confident that 3D accelerometry and the derived value of ODBA detected all flapping bouts. The distinction between soaring and gliding flight was easily derived from the flight paths of the birds: as soon as birds started to fly in circles and have a positive vertical speed (i.e. starting climbing), they were considered as “soaring”, otherwise “gliding” when the flight path was rectilinear and altitude was decreasing [42].

In the raw ECG signal, we identified the PQRS complex (i.e. the complex of electric signals defining a heartbeat, see [17]) by automatically extracting the R peak of each complex. Only peaks positively identified, by visual inspection, as being part of the PQRS complex were kept for analysis, while noise signal from muscle activity was discarded and HR was calculated as inversed R–R interval [17].

HR and ODBA were averaged every minute from 10 min before take-off until 10 min after landing (the notation adopted was related to take off (TO) or landing (LD) and minutes before or after: before flight (TO-10 to TO-1), at take off (TO), during flight (TO+1 to TO+17), upon landing (LD-2 to LD+1) and after landing (LD+2 to LD+10)). On land, we distinguished “perching” and “resting” behaviours based on the atmosphere around the birds. Both perched and resting birds were on their “perching roost”, but tourists made for a noisy atmosphere as the show was going on. In contrast, a resting bird was in a sleeping state during the morning hours when tourists were absent and the atmosphere silent. We defined the “baseline” HR as the value when birds were “resting” and considered it to be a state near Field Basal Metabolic Rate.

We considered HR as a proxy for metabolism since HR measured in a resting Eurasian griffon vulture as well as other birds is correlated with oxygen consumption [29] [16]. We collected data for a total of 255 min of flight but variability in HR patterns, inter- and intra-individual variations could not be statistically assessed with only 2 individuals. We thus presented only mean ± SD values.

### d) Ethics statement

The study was not specifically approved by an ethical committee as a permit for equipping vultures with loggers was provided as part of the licence of O. Duriez from the Research Centre for Bird Population Studies (CRBPO) of the Natural History Museum (MNHN, Paris). According to the French law of 22 September 2008, the CRBPO has the delegation by the Ministry of Ecology, Energy, Sustainable Development and Land Settlement for allowing the owners of a general bird ringing licence to capture and handle birds from protected species and mark them (with rings or any devices like loggers). The study was conducted under a formal agreement between the animal rearing facility (Rocher des Aigles) and CNRS. Birds were handled by their usual trainer, under the permit of the Rocher des Aigles (national certificate to maintain birds “Certificat de capacité” delivered to the director, Raphaël Arnaud on 4 November 1982). Care was taken to minimize discomfort to the birds and loggers were removed promptly after flights.

## Results

**Figure 1:**
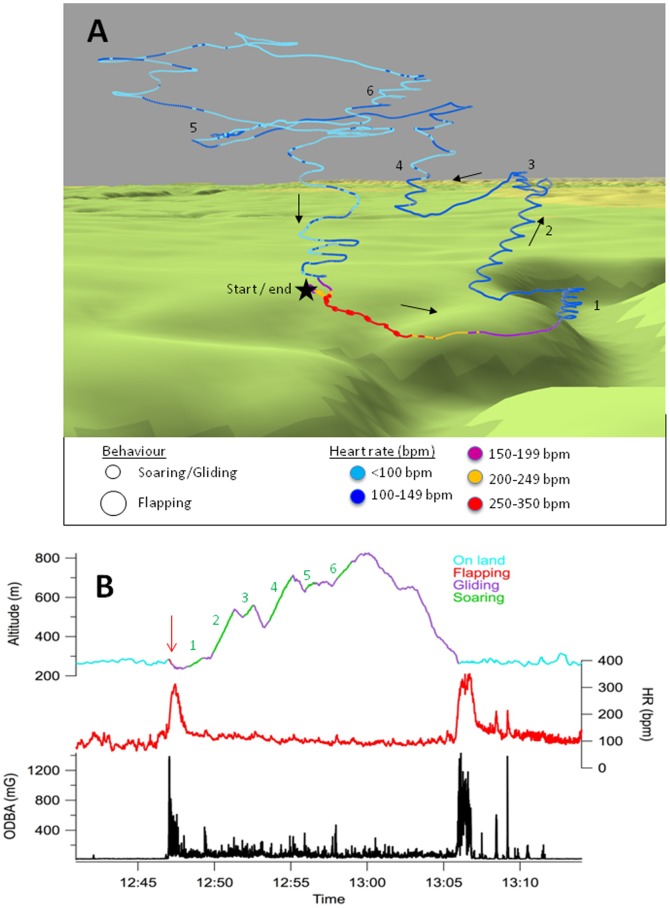
Example of flight track, heart rate and behaviour. A) One of the flight paths followed by the Himalayan griffon vulture under sunny conditions, with information on HR (gradient of colour from red (HR>300 bpm), orange, purple, to blue (HR<100 bpm)) and behaviour (small dots  =  soaring/gliding; large dots  =  flapping) superimposed. The black star marks the start and end of flight and black arrows give the first direction of flight. Soaring phases are numbered in black for an easy correspondence with panel B (see also Fig. S1 for 3-D visualization); B) a time-based projection of altitude (top), heart rate (HR, middle, in red) and an index of body activity based on acceleration signals, highlighting the flapping and walking bouts (ODBA, bottom, in black). For the altitude panel, the colour of the line changes with behaviour (with the flapping bout marked by a red arrow) and the green numbers refer to the same soaring phases as in panel A.

**Figure 2:**
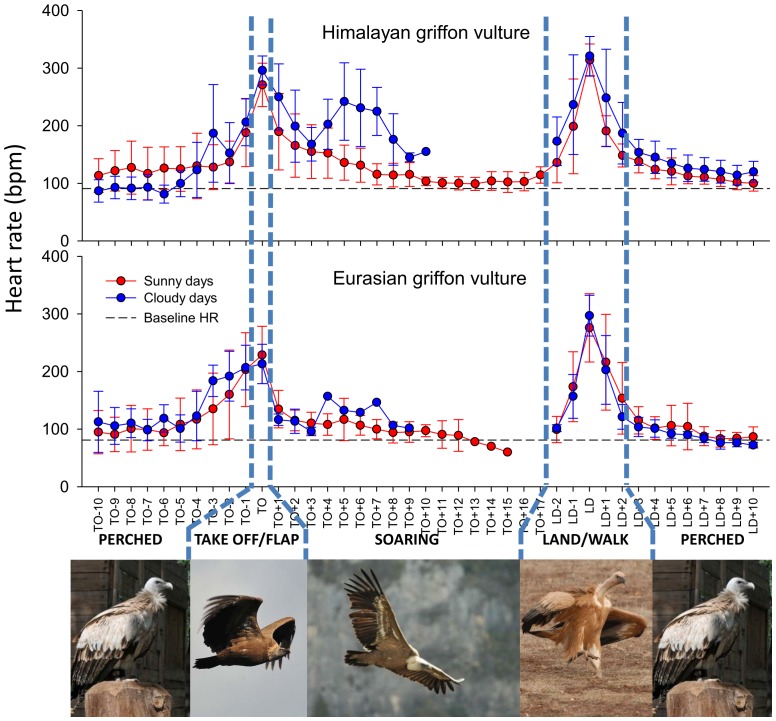
Variation of mean heart rate with weather conditions. Mean heart rate (± SD) calculated every minute, before flight (TO-10 to TO-1), at take off (TO), during flight (TO+1 to TO+17), upon landing (LD-2 to LD+1) and after landing (LD+2 to LD+10) for two griffon vultures, in sunny (red) and cloudy days (blue). The horizontal line represents baseline HR (when perched).

The lowest “resting” HR values were recorded in early morning when the birds were at rest (44.6±3.5 and 52.2±5.5 beats per minute (bpm), for Eurasian and Himalayan griffons, respectively). When vultures were perched and vigilant in the noisy atmosphere preceding flights, the baseline HRs were higher (Eurasian griffon  =  81.0±23.5 and Himalayan griffon  =  91.2±23.5 bpm; Figs 1 and 2). Approximately 3 minutes before the flight, the HR increased to >150 bpm. This corresponded to the warming up phase when vultures usually open their wings, and sometimes shake them vigorously on their perch. At take-off, vultures flapped intensively for the first minute of flight, with a mean associated HR of 222±43 bpm (max. 382 bpm) and 281±34 bpm (max. 378 bpm) for Eurasian and Himalayan griffons, respectively. Once soaring in an ascending current, HR dropped rapidly, and after 10 min of flight, HR reached near baseline values again (Eurasian griffon  =  81.1±19.1 bpm (range 60.1–108.5 bpm) and Himalayan griffon  =  103.5±13.3 bpm (range 88.2–134.3 bpm); i.e. 1 and 1.13 times baseline HRs for Eurasian and Himalayan griffons, respectively) (Figs 1, 2 and S1). Note that the soaring and gliding phases occasionally included a few short flapping bouts, depending on weather and the need for sudden acceleration by the bird. The maximum flight heights above ground reached by the two vultures were 657 m for Eurasian griffon and 825 m for Himalayan griffon (Table 1).

Less than 1 min before landing, HR increased again (Eurasian griffon  =  166±51 and Himalayan griffon  =  214±81 bpm) reaching ca. 300 bpm by the time of landing (Eurasian griffon  =  284.7±49.9 bpm (range 199–350 bpm) and Himalayan griffon  =  316.8±28.9 bpm (range 278–363 bpm)). After landing, vultures walked or ran towards their perch, displaying high HR (Eurasian griffon  =  211.1±78.8 and Himalayan griffon  =  210.0±55.2 bpm).

**Figure 3:**
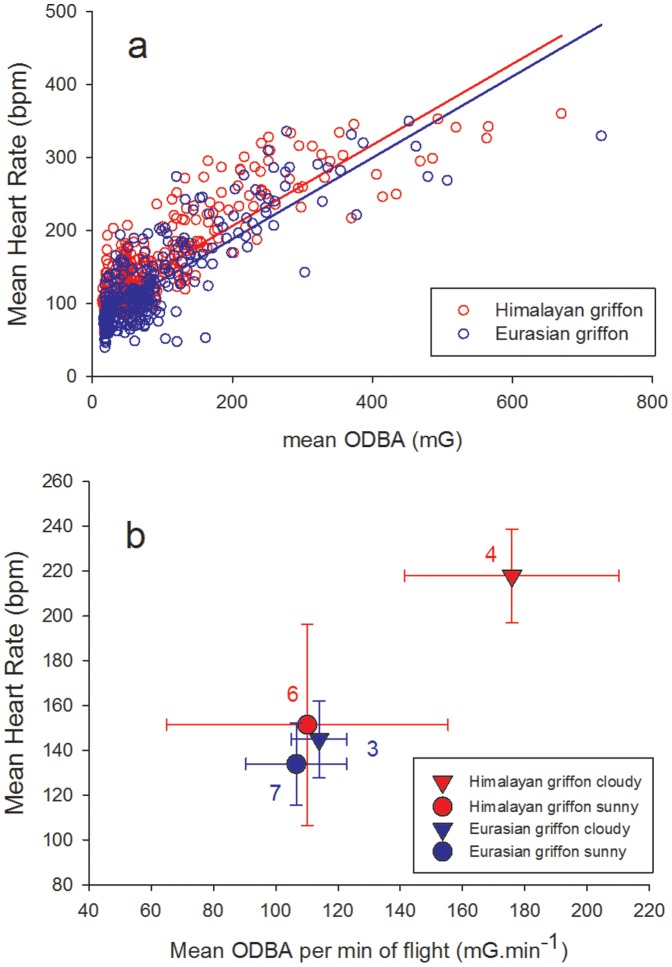
Correlation between heart rate and ODBA. Correlation between mean ± SD heart rate and Overall Dynamic Body Acceleration (ODBA) for the Himalayan (Himalayan griffon, red) and Eurasian griffon vultures (Eurasian griffon, blue): a) values averaged over one minute (including data from flight and from land (from TO-10 to LD+10); n = 382 min for Himalayan griffon and 337 min for Eurasian griffon). Regression lines are drawn in red for Himalayan griffon (R^2^ = 0.729) and in blue for Eurasian griffon (R^2^ = 0.688). b) values averaged over complete flights (TO to TO+17; no data from land), separating sunny (circles, 13 flights: 6 for Himalayan griffon and 7 for Eurasian griffon) and cloudy days (triangles; 7 flights: 4 for Himalayan griffon and 3 for Eurasian griffon).

Average HR and ODBA values calculated each minute for the whole recordings (i.e. from TO-10 to LD+10) were significantly correlated for both birds (R^2^ = 0.729; F _1,380_ = 1023.3, p<0.001 for Himalayan griffon and R^2^ = 0.69, F _1,335_ = 741.6, p<0.001 for Eurasian griffon; Fig. 3a). The slopes of the regression line were identical in both individuals (HR = 0.55*ODBA + 95.8 for Himalayan griffon; HR = 0.55*ODBA + 77.2 for Eurasian griffon). This correlation is also illustrated by the transitory increase in HR simultaneously with each bout of wing beat activity (Fig. 1B and S2).

Weather conditions influenced vulture flight. Under cloudy conditions birds, tended to make shorter flights, reaching lower altitudes (Table 1). However each individual bird behaved differently in cloudy conditions. The Himalayan griffon spent more time flapping and made shorter soaring bouts with increased ODBA during soaring. These parameters did not change between sunny and cloudy conditions in Eurasian griffon (Table 1). Consequently overall mean ODBA and HR increased by 60% and 44% respectively in cloudy conditions compared to sunny conditions in Himalayan griffon while they only marginally increased (6–8%) for Eurasian griffon (fig 3b). HR decreased faster and reached lower values in sunny conditions than under clouds in Himalayan griffon (Fig 2).

## Discussion

Our main result was very low HR values, presumably reflecting the low energy cost of soaring and gliding flight in vultures, comparable to perching birds. Resting HR values of 40–50 bpm, measured in calm conditions during the morning, were in the range of resting HR values measured under standard conditions at night in Eurasian griffon vultures [29]. Thus, the metabolic rate when resting, as derived from the HR recorded here, should be close to BMR values. Bahat [29] described a linear relationship (1) between HR (in bpm) and oxygen consumption (VO_2_, in l h^−1^ kg^−1^) in Eurasian griffon vulture resting and walking on treadmill in a respirometer chamber: 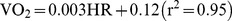
(1)


Assuming that the latter relationship is also true for flight, and assuming that 1 litre O_2_ has an energy equivalent of 20.112 KJ [43], the energy expenditure (in W kg^−1^) for Eurasian griffon vulture in gliding flight (HR of 81.1±19.1 bpm) may be converted to 2.03±0.99 W kg^−1^. With energy expenditure at rest of 1.42±0.73 W kg^−1^ (assumed to be BMR), Eurasian griffon vulture flight would represent 1.43× BMR. When applying the same conversions for energy expenditure at take-off, landing and running (respectively 4.39±1.39 W kg^−1^; 5.44±1.51 W kg^−1^ and 4.21±1.99 W kg^−1^), all these behaviours would represent energy costs ≥3× BMR. Such a low cost of flight is in accordance with theoretical predictions of 1.5× basal HR [1] but our data represent the first attempt to estimate flight costs in freely-flying raptors.

Although the relationships between ODBA and metabolic rate may be modified in different media [36], [44] (e.g. land vs air in our case), ODBA is a good predictor of energy expenditure in various vertebrate taxa [35], [36], [44]–[46]. Recently, Elliott et al. [37] showed that for thick-billed guillemots *Uria lomvia* using three different locomotory modes in different media (swimming in water, flying in air and walking on land), dynamic body acceleration was a good overall predictor of daily energy expenditure measured independently using doubly-labelled water. In this context and assuming a linear relationship between HR, ODBA (as we measured it) and metabolic rate, soaring and gliding vultures may use a similar amount of energy as when perched and 2 to 3 times less energy than when flapping or walking.

Similar results were also reported for albatrosses that use dynamic soaring flight [4], [43] and smaller birds such as gulls and bee-eaters, that frequently use soaring-gliding flight but in shorter bouts [20], [23]. Yet in some species that alternate soaring and gliding flights in short bouts, like Cape gannets *Morus capensis*, HR during gliding phases is only reduced by 20% compared to that during flapping phases [17]. Hence gannets likely save little energy when gliding. In contrast, specialized gliders, such as vultures, may save a lot of energy (>60%) when soaring and gliding, and flapping is really uncommon in vultures (generally <5% of flight time, see table 1) compared with gannets.

Another striking result was the amplitude and speed of the changes in HR; it took only a few minutes for vulture HRs to stabilize after flapping ceased. This was unexpected given that HRs of other soaring birds like the wandering albatrosses *Diomedea exulans*, which glides over the sea. The HR of these birds can take up to two hours to return to baseline levels [4]. In contrast, marine birds that rely on intensive flapping, like Cape gannets, only take a few seconds to decrease and increase their HR at the start of a gliding and a flapping session, respectively, though the actual difference is smaller and each session is shorter [17].

Each bird reacted differently to the presence of cloud cover. The predicted increase in HR during cloudy conditions was observed for the Himalayan griffon but only hardly affected Eurasian griffon, as compared to sunny days when soaring flight was likely easier due to the occurrence of stronger thermals. This was when birds engaged in longer soaring-gliding bouts and highlights the importance for vultures of waiting for adequate weather conditions to soar efficiently [7] and how they must “harvest energy” from the landscape when searching for updrafts [42]. The larger wingspan of Himalayan griffon could explain its greater sensitivity to cloudy conditions as flapping should be more costly for larger birds [3], while its lower wing loading would allow a more economic use of thermals (less flaps) in sunny conditions. Still the fact that Eurasian griffon was able to maintain its effort relatively constant in cloudy days compared to sunny days was surprising. Perhaps the temperate living Eurasian griffon more commonly flies during cloudy conditions with relatively low wind speeds compared to the Himalayan griffon which lives in tropical mountains where wind speeds are higher? Or does this result stem from the difference in experience of the birds with the older bird (Eurasian griffon) having better knowledge of the best site to find an ascending current than the younger Himalayan griffon?

Although necessarily preliminary with regards to the small sample size, our results confirm that soaring and gliding flights are relatively cheap modes of travel and provide the first attempt to quantify the cost of this locomotory mode. Since vultures can fly >200 km daily [47]–[49], and because their scavenging diet should favour physiological and behavioural adaptations to preserve energy [28], we expected vultures to develop specific adaptations to reduce costs at minimum during flight, which is likely the most energetically expensive behaviour they undertake. As flight accounts for >50% of the daily activity budget of free-ranging vultures [50], reductions in heart rate thus represent substantial energy savings. On the other hand, the elevated HR recorded when taking-off and landing as well as walking suggests that feeding events (involving alternation of short walks, runs, jumps and fights [26], [51]) must be very costly for vultures. As an illustration of this, vultures seem to physiologically “prepare” their body before the strenuous effort to take off for flight by shaking their wings which is accompanied by an increase in HR a few seconds before take-off. This resembles the increase in HR before take-off described in greater white-fronted geese *Anser albifrons* that shake head before flight [52]. Similarly, just before landing, the increase in HR is certainly mostly related to the fine adjustments of the tail, wings and legs, required to control of the trajectory and to reduce speed in order to land softly [53], [54]. However this increase in HR may also reflect a preparation of the body to the effort to come on land, as it happened up to 2 min before the final flaps and landing. Because vultures feed in groups but must compete to access carrion [51], [55], such high cost for landing would explain why sometimes vultures prefer to stay aloft rather than landing at a carcass, when their chance to access food is limited. Also, the increase in energetic costs associated with flight with distance covered, as commonly assumed for soaring birds like vultures [49] may be very limited, since we found that vultures expend nearly negligible greater amounts of energy when flying than when perching.

## Supporting Information

Figure S1 **Sample flight path.** 3-D position during one flight of the Himalayan vulture (same as in Fig 1) with superimposed information on heart rate (gradient of colour from red (HR>300 bpm), orange, purple, to blue (HR<100 bpm)) and behaviour (small triangles  =  soaring; small dots  =  gliding; large dots  =  flapping). The KML file is viewable with Google Earth software (http://www.google.com/intl/en/earth/index.html). A click on each dot displays time, altitude, HR, ODBA and behaviour.
(ZIP)

Figure S2 **Acceleration, ODBA and Heart rate.** Example of the first minute of one flight of Eurasian griffon vulture, showing the concordance between Heart rate (red line on top, in bpm), 3-D acceleration data (heave in blue, surge in red, sway in green, in mG, middle graph), and derived ODBA (in mG, black in 100 Hz and red line in 1 Hz, bottom graph). Each single wingbeat is determined by a peak on the heave and surge accelerations and the corresponding flapping bouts are shown as black rectangles above acceleration data.
(TIF)
