# Supplementary figures and images for: How Cheap Is Soaring Flight in Raptors? A Preliminary Investigation in Freely-Flying Vultures
Source: PLoS One. 2014 Jan 15;9(1):e84887. doi: 10.1371/journal.pone.0084887 (PMC3893159; doi:10.1371/journal.pone.0084887)

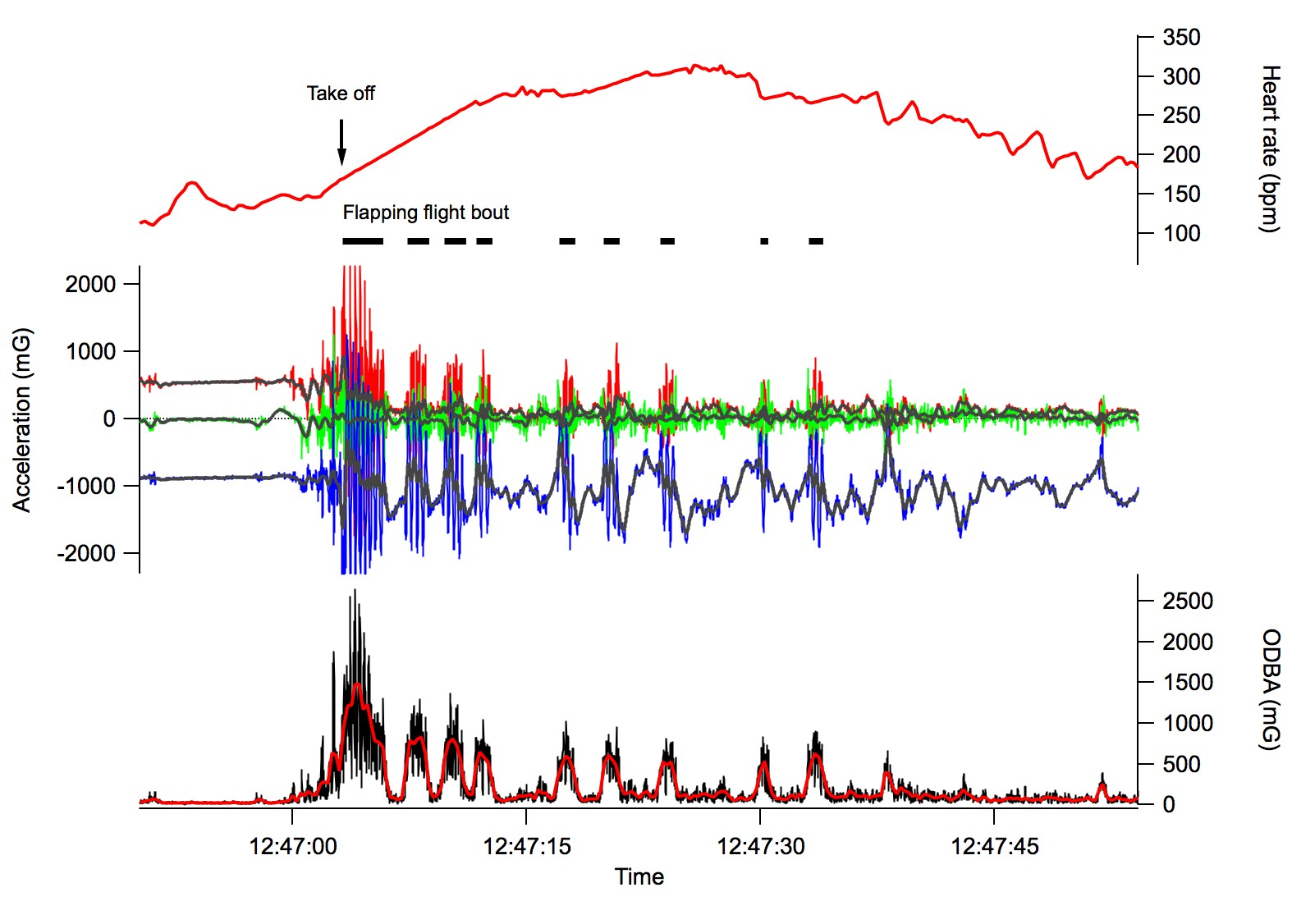

Supplement: Figure S2 — Acceleration, ODBA and Heart rate. Example of the first minute of one flight of Eurasian griffon vulture, showing the concordance between Heart rate (red line on top, in bpm), 3-D acceleration data (heave in blue, surge in red, sway in green, in mG, middle graph), and derived ODBA (in mG, black in 100 Hz and red line in 1 Hz, bottom graph). Each single wingbeat is determined by a peak on the heave and surge accelerations and the corresponding flapping bouts are shown as black rectangles above acceleration data. (TIF) [file pone.0084887.s002.tif]
